# Folding mechanisms steer the amyloid fibril formation propensity of highly homologous proteins†
†Electronic supplementary information (ESI) available. See DOI: 10.1039/c8sc00166a


**DOI:** 10.1039/c8sc00166a

**Published:** 2018-03-01

**Authors:** Gaetano Malgieri, Gianluca D'Abrosca, Luciano Pirone, Angelo Toto, Maddalena Palmieri, Luigi Russo, Michele Francesco Maria Sciacca, Rosarita Tatè, Valeria Sivo, Ilaria Baglivo, Roksana Majewska, Massimo Coletta, Paolo Vincenzo Pedone, Carla Isernia, Mario De Stefano, Stefano Gianni, Emilia Maria Pedone, Danilo Milardi, Roberto Fattorusso

**Affiliations:** a Department of Environmental, Biological and Pharmaceutical Sciences and Technologies , University of Campania “Luigi Vanvitelli” , Via Vivaldi 43 , 81100 Caserta , Italy . Email: roberto.fattorusso@unicampania.it; b Institute of Biostructures and Bioimaging , CNR , Via Mezzocannone 16 , 80134 Naples , Italy; c Department of Biochemical Sciences “Alessandro Rossi Fanelli” , University of Rome “La Sapienza” , Piazzale Aldo Moro 5 , 00185 , Roma , Italy; d Institute of Biostructures and Bioimaging , CNR , Viale A. Doria 6 , 95125 Catania , Italy . Email: dmilardi@unict.it; e Institute of Genetics and Biophysics “Adriano Buzzati-Traverso” , CNR , Via P. Castellino 111 , 80131 Napoli , Italy; f Department of Clinical Sciences and Translational Medicine , University of Rome “Tor Vergata” , Via Montpellier 1 , 00133 , Roma , Italy

## Abstract

Significant advances in the understanding of the molecular determinants of fibrillogenesis can be expected from comparative studies of the aggregation propensities of proteins with highly homologous structures but different folding pathways. Here, we fully characterize, by means of stopped-flow, T-jump, CD and DSC experiments, the unfolding mechanisms of three highly homologous proteins, zinc binding Ros87 and Ml1_53–149_ and zinc-lacking Ml4_52–151_. The results indicate that the three proteins significantly differ in terms of stability and (un)folding mechanisms. Particularly, Ros87 and Ml1_53–149_ appear to be much more stable to guanidine denaturation and are characterized by folding mechanisms including the presence of an intermediate. On the other hand, metal lacking Ml4_52–151_ folds according to a classic two-state model. Successively, we have monitored the capabilities of Ros87, Ml4_52–151_ and Ml1_53–149_ to form amyloid fibrils under native conditions. Particularly, we show, by CD, fluorescence, DLS, TEM and SEM experiments, that after 168 hours, amyloid formation of Ros87 has started, while Ml1_53–149_ has formed only amorphous aggregates and Ml4_52–151_ is still monomeric in solution. This study shows how metal binding can influence protein folding pathways and thereby control conformational accessibility to aggregation-prone states, which in turn changes aggregation kinetics, shedding light on the role of metal ions in the development of protein deposition diseases.

## Abstract 2

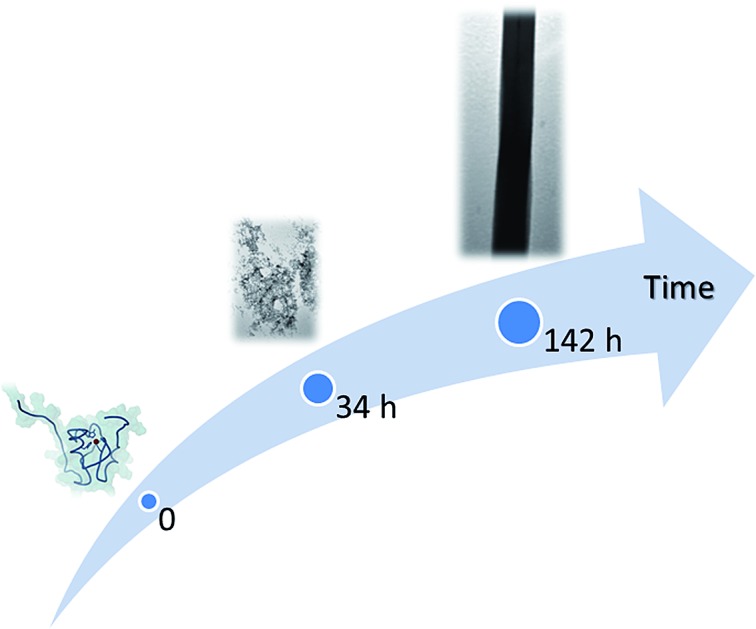
Understanding the molecular determinants of fibrillogenesis by studying the aggregation propensities of high homologous proteins with different folding pathways.

## Introduction

Amyloid fibrils and plaques are the characteristic traits of various degenerative conditions affecting both the central nervous system and/or several peripheral tissues. These conditions include Alzheimer's, Parkinson's, prion diseases and numerous forms of fatal systemic amyloidosis, collectively known as “protein misfolding diseases”.^1^–^3^ Despite intensive research efforts focusing on these themes in the last few decades, the molecular mechanism by which proteins associated with these diseases aggregate to form amyloid structures remains partly unveiled. Many proteins can adopt metastable, partially structured conformations stabilized by oligomerization prior to forming amyloid fibrils.^2^ In these amyloid prone-states, proteins can nucleate initial oligomeric assemblies where the content of β-sheet secondary structures is generally increased. These “seeds” or “nuclei” provide a sort of template where other misfolded or partially folded molecules are recruited, thereby increasing the size of the assemblies that finally give rise to fibrils.^4^ Protein misfolding disorders are caused, in most cases, by the aggregation of unstructured peptides, intrinsically disordered proteins and fragments of otherwise structured proteins. However, globular proteins, having persistent and well defined secondary and tertiary structures, have also been described to undergo aggregation causing amyloid diseases.^3^ It is clear that the possibility to form amyloid structures is not an exclusive mark of proteins directly associated with neurodegenerative diseases but is an inherent property in all polypeptide sequences.^1^ In the cellular environment the onset of aggregation may be triggered by any factor that results in a rise of concentration of the amyloidogenic precursor, thereby shifting the equilibrium between correctly and partially folded molecules.^2^ Protein mutations, environmental changes, chemical modifications and metal ions have been reported to influence amyloid formation.^1^,^5^,^6^ The analysis of how these factors impact protein folding pathways may provide decisive clues about the propensity to form aggregates and fibrils.^1^,^7^,^8^ However, more significant advances in the understanding of the molecular determinants of fibrillogenesis are, in principle, expected from comparative studies of the aggregation propensities of proteins with highly homologous structures but different folding pathways.

**Fig. 1:**
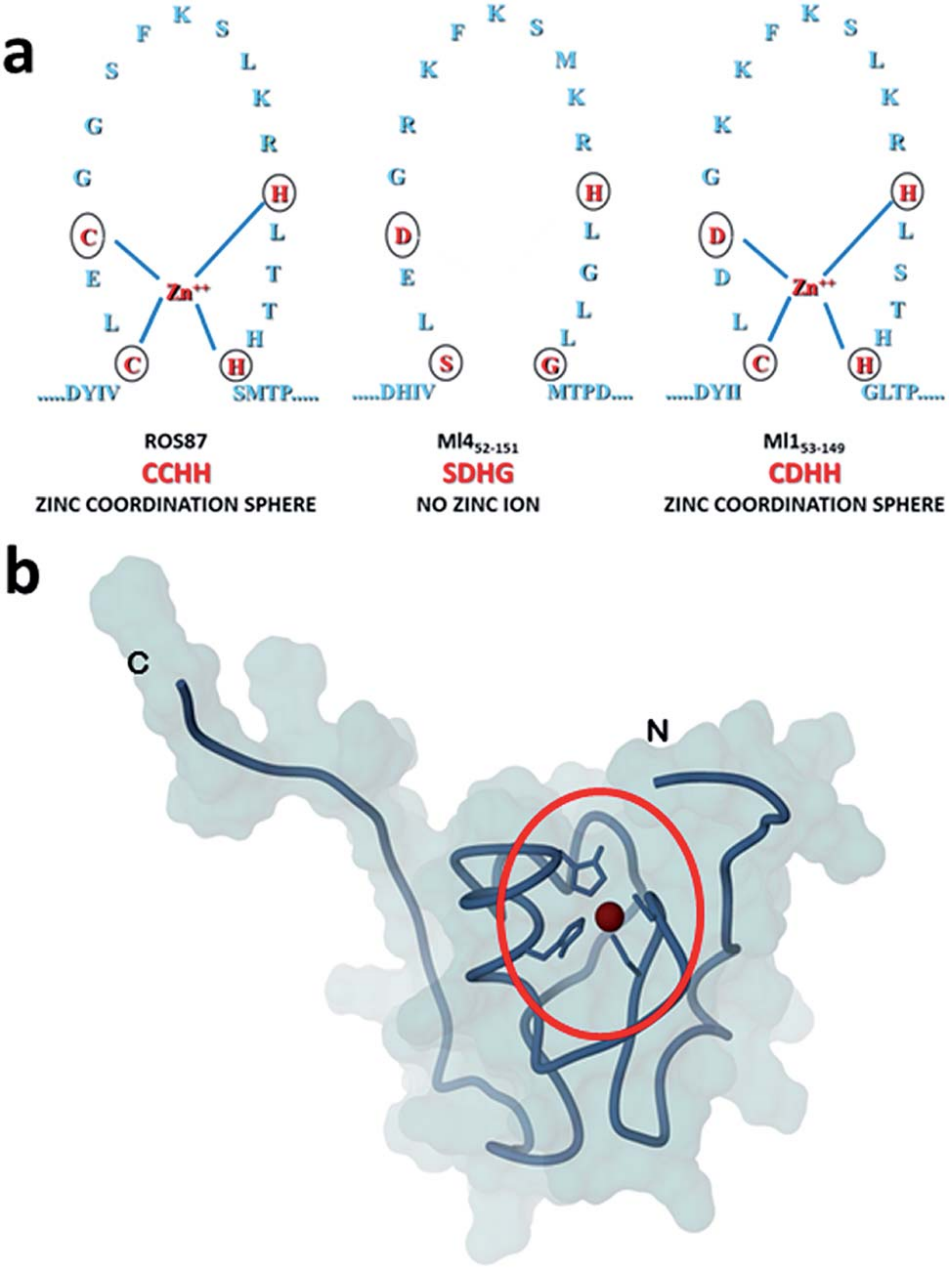
Coordination spheres. (a) Circles indicate positions corresponding to the coordination residues in Ros87 and Ml1_53–149_. Ml4_52–151_ does not bind the structural Zn^2+^. (b) The globular fold of Ros87;^10^ the circle evidences the Zn^2+^ coordination sphere.

Recently, we have characterized the folding mechanisms of DNA binding domains of two highly homologous proteins belonging to the prokaryotic zinc finger family.^8^ Particularly, metal-free Ml4_52–151_ folds *via* a classic two-state cooperative transition while the metal-binding structural homologue, Ros_56–142_ (hereafter Ros87), exhibits a complex folding pathway featuring a well-defined metal-binding intermediate, whose transition to the native state involves a delicate barrier-less downhill scenario. Therefore, members of this class of proteins^9^–^15^ may represent a valuable paradigm to study the causative relationship linking different protein folding scenarios with fibrillogenesis. To this aim, here we integrate, by means of kinetic and equilibrium experiments, the characterization of the unfolding mechanism of Ros87 and Ml4_52–151_ and extend the study to Ml1_53–149_, a structural homologue which binds Zn^2+^ through a coordination sphere different from that of Ros87 (Fig. 1). We then compared the amyloidogenic propensities of the three isostructural proteins to form amyloid fibers under different temperature and pH conditions by circular dichroism (CD) and fluorescence spectroscopies, dynamic light scattering (DLS), and transmission and scanning electron microscopies (TEM and SEM^16^). The results obtained indicate that different folding mechanisms may strongly influence amyloid fibril formation in highly homologous proteins.

## Experimental section

### Proteins and fibril preparation

All the proteins were recombinantly expressed in *E. coli* and purified as previously described.^10^,^11^ Gel electrophoresis and mass spectrometry were used to verify the identity and purity of the proteins.

For fibrillation studies, 100 μM or 300 μM purified proteins in 20 mM phosphate buffer (pH = 6.8) and 0.2 M NaCl were incubated at 298 K (Ros87, Ml4_52–151_, and Ml1_53–149_) or 288 K (Ml4_52–151_). The samples under acidic conditions (pH = 3) were obtained by adding aliquots of 0.1 M HCl to freshly prepared protein solutions.

### Stopped-flow

Unfolding and refolding kinetic experiments were carried out using an SX-18 stopped-flow apparatus (Applied Photophysics, Leatherhead, UK). Experiments on Ros87 and Ml1_53–149_, were conducted at 298 K, in 20 mM sodium phosphate, 300 mM NaCl, 100 μM ZnCl_2_ and 4 mM TCEP at pH 6.8 and different concentrations of GdnHCl. For Ml4_52–151_ we used the same experimental conditions with the addition of 0.4 M Na_2_SO_4_ to the buffer. At least 5 individual traces were acquired and then averaged for each experiment. The final protein concentration was typically 2 μM for Ros87 and Ml4_52–151_, and 1 μM for Ml1_53–149_. Protein samples were excited at 280 nm and fluorescence emission was measured using a 320 nm cut-off glass filter.

### T-jump

Relaxation unfolding experiments of Ros87 were conducted using a Hi-Tech PTJ-64 capacitor-discharge T-jump apparatus (Hi-Tech, Salisbury, UK). The buffer used was 20 mM sodium phosphate, 300 mM NaCl, 100 μM ZnCl_2_, and 4 mM TCEP at pH 6.8. Temperature was rapidly changed with an increase of 9 K, from 289 to 298 K. Experiments were performed in solutions containing different GdnHCl concentrations. Degassed samples were slowly pumped through a 0.5 mm × 2 mm quartz flow cell before acquisition of data, and then 10–20 individual traces were acquired and averaged. The excitation wavelength was 296 nm, and the fluorescence emission was measured using a 320 nm cut-off glass filter.

### Equilibrium experiments

Equilibrium unfolding experiments on Ros87, Ml1_53–149_ and Ml4_52–151_ were carried out using a Fluoromax single photon counting fluorometer (Jobin-Yvon, NJ, USA) by mixing the native proteins, at a concentration of 2 μM for Ros87 and Ml4_52–151_, and 1 μM for Ml1_53–149_, with increasing concentrations of GdnHCl. The experiments were performed using 20 mM sodium phosphate, 300 mM NaCl, 100 μM ZnCl_2_, and 4 mM TCEP at pH 6.8 (with the addition of 0.4 M Na_2_SO_4_ for Ml4_52–151_ experiments) and at a temperature of 298 K. Samples were excited at a wavelength of 280 nm and the intrinsic tryptophan fluorescence was recorded between 300 and 400 nm.

### ThT fluorescence

Samples were prepared by adding 10 μM ThT to 100 μL of protein solution with a final protein concentration of 100 μM. To detect amyloid formation, fluorescence spectra were collected at 298 K (or 288 K for Ml4_52–151_) using a Varian Cary Eclipse spectrophotometer and a 0.1 cm path length cell. ThT fluorescence emission spectra were acquired in the range 450–600 nm upon excitation at 440 nm. Both excitation and emission slits were set at 10 nm. Fluorescence intensity values at 520 nm were plotted as a function of time. In the case of unfolded Ros87 and samples with a protein concentration of 300 μM, experiments were carried out in Corning 96 well non-binding surface plates. 5 day time traces were recorded using a ThermoFisher Varioskan plate reader at excitation and emission wavelengths of 440 nm and 480 nm, respectively, at 298 K, shaking samples for 10 seconds before each reading.

### Dynamic light scattering (DLS)

DLS measurements were carried out using a Malvern nano zetasizer (Malvern, UK). The proteins (100 μM) were incubated at 298 K (or 288 K for Ml4_52–151_) over time. Samples were placed in a disposable cuvette and held at the corresponding temperature during the analysis. The aggregation rate was monitored from time 0 until 7 days of incubation. For each sample, the analyses were recorded three times with 11 sub-runs using the multimodal mode. The *Z*-average diameter was calculated from the correlation function using Malvern technology software.

### Circular dichroism spectroscopy (CD)

A JASCO J-815 CD spectropolarimeter equipped with a Peltier temperature controller has been used to collect CD spectra with a data pitch of 1 nm, using a quartz cuvette with a 1 cm path length in the 200–260 nm wavelength range. All data were recorded with a scanning speed of 50 nm min^–1^ and a band width of 1 nm. Data were normalized against reference spectra to remove the background contribution of buffer. During the melting experiments, CD spectra were collected at 3 K intervals in the 278–383 K range. At the end of measurements, the samples were cooled back to 298 K and a final set of spectra was collected. The experiments were conducted on 25 μM Ml1_53–149_ protein. A two-state folding model^17^ has been used to fit the obtained data.

### Differential scanning calorimetry (DSC)

DSC experiments were performed by using a MicroCal VP-DSC calorimeter. After degassing, all protein samples were heated at 1 K min^–1^ in the temperature range 293–373 K. To prevent the formation of air bubbles during heating, an extra ∼29 psi external pressure was applied. Several buffer–buffer heating scans were regularly performed before the measurement in order to properly equilibrate the calorimeter. Protein scans were recorded only after obtaining invariant buffer–buffer baselines. To exclude uncontrolled drifts in the instrumental baseline, extra buffer–buffer baselines were obtained directly after the protein scans. Three independent DSC experiments were recorded at different protein concentrations within the 60–250 μM range using the same buffer conditions. The heat capacity *C*_p_ curves have been obtained by recording buffer–buffer baselines at the same scan rate and subtracting them from sample curves, as previously described.^8^,^18^ One or several heating–cooling cycles were completed to determine the reversibility of the denaturation process. Software supplied by MicroCal as previously described^19^ has been used to calculate the experimental values of the absolute heat capacity of Ml1_53–149_ at different protein concentrations from the experimental thermograms. The partial specific volume of Ml1_53–149_ was calculated using the equation reported by Fischer *et al.*^20^ The native baseline could not be extrapolated from the DSC thermogram. We have employed a previously proposed empirical equation in which the heat capacity *versus T* function is directly calculated from the molecular mass of the protein.

In the formalism adopted to describe downhill folding by DSC,^21^ Σ*α* roughly corresponds to the difference in enthalpy between the thermodynamic states populated at low and high temperatures. *β* may be related to the energy barrier which separates protein macrostates. Of course, very small positive *β* values will be essentially equivalent to the barrierless (downhill) case.

### Transmission electron microscopy (TEM)

For TEM analysis, suspensions of proteins collected at regular time points were applied to carbon-coated copper grids, blotted, washed, negatively stained with 2% (w/v) of phosphotungstic acid, air dried and then examined with a Philips CM12 transmission electron microscope operating at 200 keV. Images were recorded photographically on Kodak type 4489 and SO163 electron image films, and digitally with a column-mounted Mega View III CCD camera. Photographic negatives were scanned using an Epson Perfection 1640SU, as ∼3 MB tif files with an image resolution of at least 600 dpi.

### Scanning electron microscopy (SEM)

Suspensions of proteins were applied to glass-coated stainless-steel grids, blotted, washed, air dried and then examined with a Hitachi S4800 scanning electron microscope operating at an accelerating voltage of 15 keV.

SEM analysis was also performed on air dried samples after fifteen days of incubation to avoid the CPD preparation procedure in order to compare the fibril images obtained.

## Results

### Characterization of folding mechanisms of Ros87, Ml1_53–149_ and Ml4_52–151_

To compare the folding mechanisms of Ros87, Ml1_53–149_ and Ml4_52–151_ (Fig. 1) we resorted to carry out equilibrium and kinetic experiments. Therefore, the three proteins were subjected to chaotropic denaturant induced (un)folding.

**Fig. 2:**
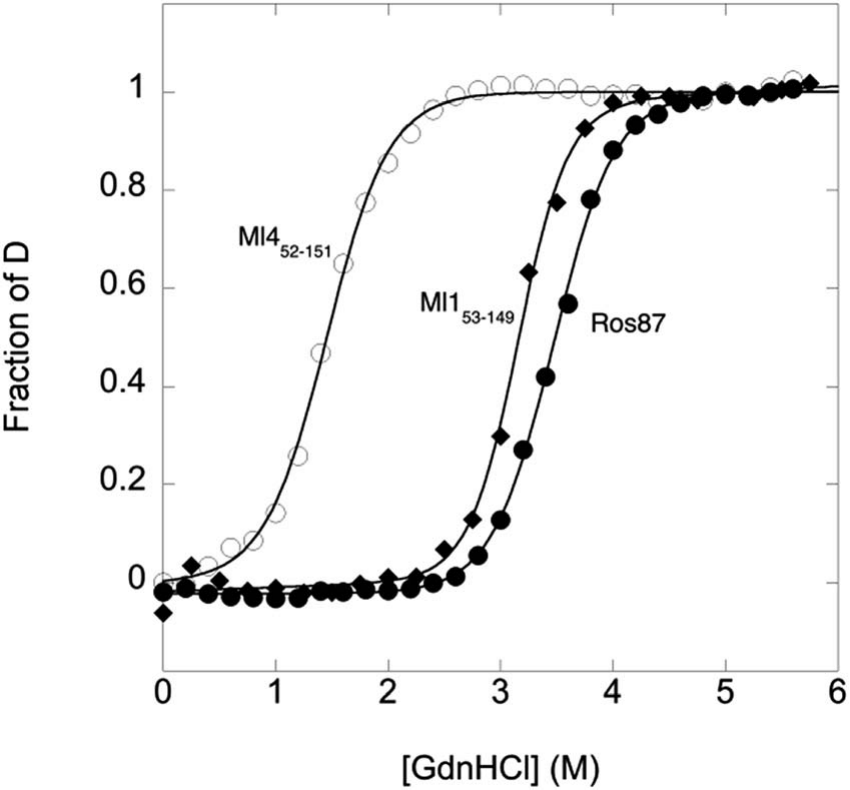
GdnHCl-induced equilibrium denaturation of Ros87, Ml1_53–149_ and Ml4_52–151_ at 298 K and pH 6.8 using 20 mM sodium phosphate, 300 mM NaCl, 100 μM ZnCl_2_, and 4 mM TCEP, as a function of intrinsic tryptophan emission.

GdnHCl-induced equilibrium denaturation of Ros87, Ml1_53–149_ and Ml4_52–151_ monitored by the decrease of intrinsic tryptophan emission, is reported in Fig. 2.

It should be noted that, due to its lower thermodynamic stability, the experiments on Ml4_52–151_ were carried out in the presence of 0.4 M sodium sulfate, an inorganic salt that is classically used to stabilize proteins. In all three cases, the observed transition seems to follow a simple two-state behavior, which may suggest the absence of stable equilibrium intermediate(s). As expected, the three proteins display a similar co-operativity correlated with changes in the accessible surface area upon unfolding.^22^

**Fig. 3:**
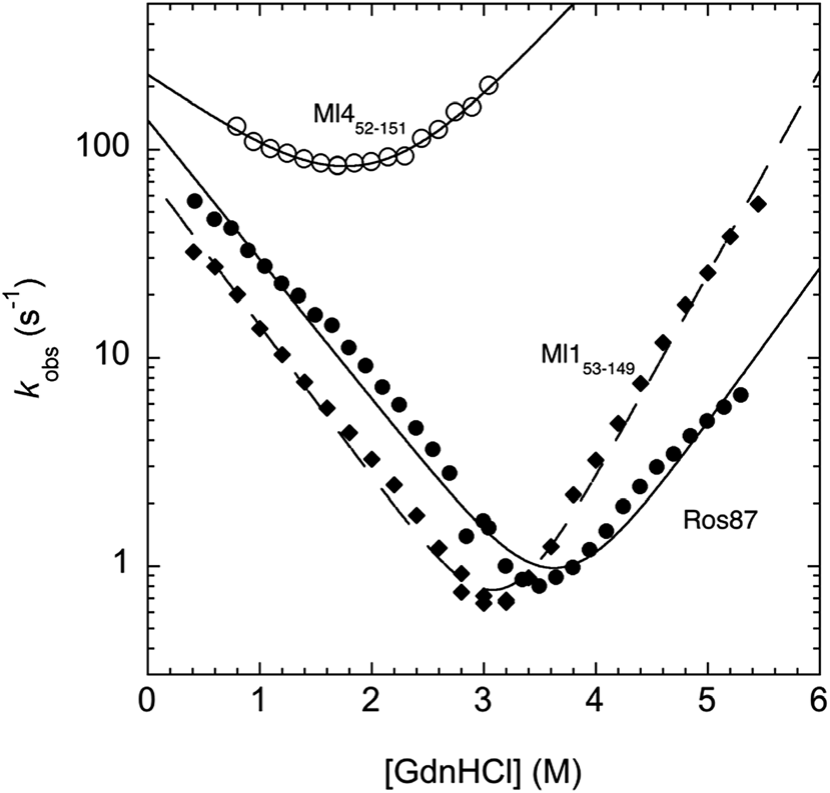
Chevron plots measured for Ros87, Ml1_53–149_ and Ml4_52–151_.

In an effort to present a complete characterization of the folding mechanisms of Ros87, Ml1_53–149_ and Ml4_52–151_ we measured the folding and unfolding kinetics of these three proteins by stopped-flow. Under all the investigated conditions, the observed kinetics were consistent with a single exponential behaviour, indicating the lack of stable folding intermediates in the ms to s time range. A semi-logarithmic plot of the observed folding/unfolding rate constant *versus* denaturant concentration (chevron plot) measured for Ros87, Ml1_53–149_ and Ml4_52–151_ is reported in Fig. 3.

Interestingly, whilst in the case of Ml4_52–151_ the logarithms of the observed refolding and unfolding rate constants decrease linearly as a function of denaturant concentration, resulting in a V-shaped chevron plot, in the case of Ros87 and Ml1_53–149_ there was an additional complexity. In fact, in the case of both proteins the observed rate constants present a downward curvature as a function of GdnHCl concentration (roll-over effects) and a clear deviation from the two-state chevron plot. This observation is classically interpreted as a signature of multistate folding and reflects the accumulation of a folding intermediate and appears to be in agreement with our earlier studies.^8^ It may be noted that, due to its low stability, the chevron plot of Ml4_52–151_ could be poorly explored. Therefore, we characterized this protein further using a milder chaotropic denaturant. Data obtained for the chevron plot of Ml4_52–151_ in urea are reported in the ESI Fig. SI1.† It may be observed that also in this case Ml4_52–151_ displays a V-shaped chevron plot, consistent with a two-state behavior.^23^ Additional support for the presence of an intermediate in the folding of Ros87 and Ml1_53–149_ comes from the comparison of the amplitudes of the observed folding and unfolding kinetic transitions. In fact, as depicted in the ESI Fig. SI2 and 3,† it may be observed that the amplitudes of the refolding traces are lower than those measured for unfolding. This effect, typically known as the burst-phase effect,^24^,^25^ is associated with the accumulation of a folding intermediate on time scales faster than those accessible by stopped-flow (*i.e.* <1–2 ms). On the basis of these observations, we conclude that whilst the folding of Ml4_52–151_ resembles a two-state mechanism, the folding of Ros87 and Ml1_53–149_ involves the presence of at least a low energy folding intermediate that folds and unfolds on an ultra-rapid time-scale.

To investigate further the fast folding phase of Ros87 and Ml1_53–149,_ we subjected the proteins to a temperature jump experiment by using a capacitor discharge instrument. In particular, solutions containing different concentrations of GdnHCl in the presence of buffer 20 mM sodium phosphate, 300 mM NaCl, 100 μM ZnCl_2_, and 4 mM TCEP were subjected to a rapid 9 K jump from *T* 289 K to 298 K. However, under all the investigated conditions, we could not detect any kinetic phase in the 30–40 μs to ms time range, indicating that the transition of the denatured state to the intermediate is either too fast for our T-jump apparatus or associated with a low change in enthalpy such that a jump of 9 K does not perturb the equilibrium sufficiently to cause a relevant shift in population between the denatured and intermediate states.

**Fig. 4:**
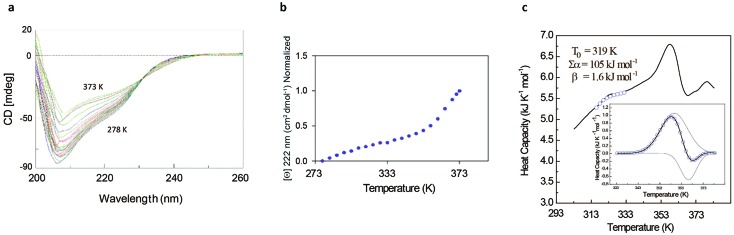
Ml1_53–149_ unfolding behavior. (a) Thermal unfolding of Ml1_53–149_ in the range 278–373 K at regular intervals of 3 K: the figure shows the isodichroic point at 233 nm observable in the range of 278–353 K. (b) Melting curve monitored by CD at 222 nm. (c) Representative profile of thermally induced unfolding of Ml1_53–149_ monitored by DSC.

The rather complex nature of the Ml1_53–149_ folding pathway was also confirmed by CD melting curves (Fig. 4a and b) as already reported for Ros87 and Ml4_52–151_.^8^ The unfolding pathway of this protein, in fact, cannot be described by using a simple two-state model (Fig. 4b). Accordingly, the thermal transition monitored by DSC consists of a first broad and reversible endotherm centred at ∼319 K followed by a second irreversible sharper endotherm centred at 360 K (Fig. 4c), suggesting the existence of intermediates which populates the thermal unfolding process. When fitting the DSC thermogram in the temperature range 303–343 K using the classic two-state formalism described by Privalov and coworkers,^26^ the obtained Van't Hoff ratio (*r*^VH^ is 1.5) is significantly far from 1.0 thus suggesting that the unfolding process cannot be described in terms of a simple two-state mechanism. This indicates that Ml1_53–149_ thermal unfolding cannot be described using a two-state model. For this reason, the DSC thermogram has been quantitatively analysed using a variable-barrier energy model proposed by Muñoz and Sanchez-Ruiz,^27^ as previously described.^8^ The inset of Fig. 4c reports a fit with a *β*-value that results in a marginal free-energy barrier (*i.e.*, ≤2*RT*), suggesting a downhill scenario. The second irreversible transition can be linked to the final metal loss,^28^ which involves a quite large enthalpy change (Δ*H* = 162 kJ mol^–1^).^29^ Analogous to Ros87, Ml1_53–149_ shows two distinct structural transitions: the recruitment of Zn^2+^ and downhill folding to the native state, which however in this case appear to be convoluted without a clear energy minimum.

These results are further supported by the NMR data (Fig. SI4†). The ^1^H–^15^N HSQC spectra acquired at different temperatures show the presence of visible cross-peaks up to 353 K, in agreement with the mechanism of folding described.

Overall, kinetic and thermodynamic characterizations clearly indicate that the folding mechanisms of these three iso-structural proteins are significantly different, providing a suitable model system to investigate the correlations between protein folding mechanisms and their amyloid formation propensities.

### Aggregation and fibrillation propensity

**Fig. 5:**
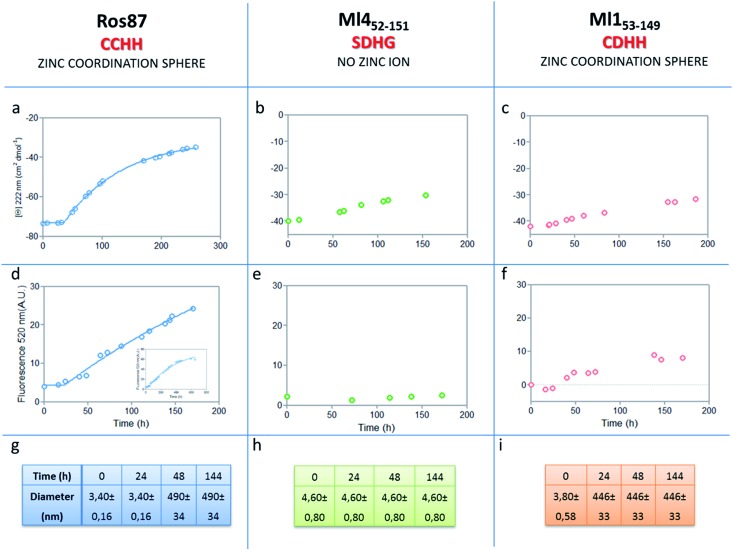
Aggregation kinetics of the three proteins. (a–c) Fitting curve^37^,^38^ of the aggregation kinetics followed by CD – (d–f) fitting of the kinetics followed by ThT assay – (g–i) the apparent hydrodynamic diameter *d* (nm) of the different proteins over time. Intensity values of DLS measurements are 100% for all the reported data.

The analysis of Ros87, Ml4_52–151_ and Ml1_53–149_ sequences performed using the server AGGRESCAN^30^ reveals that the stretch of amino acids encompassing the regions His76–Cys82 in Ros87, corresponding to Pro72–Asp79 in Ml1_53–149_ and Pro74–Glu81 in Ml4_52–151_ (Fig. SI5†), are sequences with high intrinsic aggregation propensities.^31^ This amino acid stretch folds in a β-strand (β2) and in a successive β-turn in folded Ros87 and, interestingly, is already structured within the zinc binding intermediate that separates the two transitions in the Ros87 unfolding pathway (Fig. SI5†). Based on this observation, we started the characterization of aggregation propensity of Ros87. In Fig. SI6A† the far-UV CD spectra of Ros87 as a function of time are reported. After a lag phase of 35 hours, a gradual variation of the native secondary structure content starts.^32^ The parallel measurements of DLS provide a good explanation for the phenomenon observed. In fact, DLS is very sensitive and perfectly suited for the detection of the rapid formation of small amounts of protein aggregates when the protein is still prevalently monomeric.^33^ A neat peak corresponding to a hydrodynamic radius of 1.7 nm was measured as the size distribution of Ros87 at time 0, which is well-matched with the size of the native monomer.^10^ However, the size distribution over time becomes characterized by larger particles. In particular, after 48 hours, in agreement with the observed CD phenomenon (Fig. 5a), large aggregates having a hydrodynamic radius of ∼250 nm arise in solution.

**Fig. 6:**
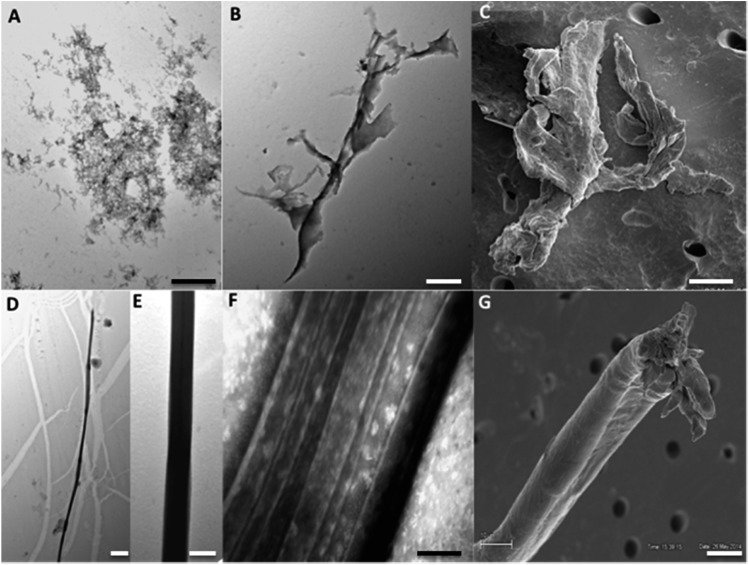
Aged Ros87 pictures. Branched protofibrils shown by TEM at 34 h (A) scale bar: 100 nm; laminar pseudo-fibrillar aggregates at 84 h (B and C), TEM image, scale bar: 200 nm (B); SEM image, scale bar: 50 nm (C); linear macrofibrillar aggregates, TEM image at 142 h, scale bar: 5 mm (D); bundles of fibrils, TEM images at 142 h (E and F), scale bar: 1 mm (E), scale bar: 200 nm (F); and linear fibrillar aggregates, SEM image, scale bar: 5 μm (G).

The ThT fluorescence analysis shows that Ros87 starts to form amyloid fibrils after ∼40 hours of incubation (Fig. SI6B† and 5d). In fact, TEM inspection after 34 hours (Fig. 6A) reveals branched protofibrils (10–15 nm diameter) which tend to aggregate into reticular clusters. After 84 hours laminar pseudo-fibrillar aggregates (approximately 80–100 nm in diameter) appear (Fig. 6B) which seem to be constituted by small bundles of proto-fibrils in SEM observations (Fig. 6C). After 142 hours, the sample shows linear macrofibrillar aggregates (Fig. 6D) with ∼1000 nm diameter constituted by bundles of fibrils ∼150–200 nm in diameter (Fig. 6E and F). SEM images of linear fibrillar aggregates (Fig. 6G) confirm the ultrastructural organization visualized by TEM. Ros87 aggregation is therefore likely to occur through conformational nucleated conversion,^34^–^36^ based on the initial formation of structured nuclei capable to seed the formation of ThT positive proto-fibrils that rapidly evolve into mature fibrils.^34^–^36^

Accordingly, to verify the intrinsic propensity of the protein sequence to form amyloid oligomers we have also studied the behavior of Ros87 under acidic conditions. Ros87 under acidic conditions is unfolded and shows, by means of ThT assays, a much faster formation of amyloid oligomers, which starts immediately without a well-defined lag phase (Fig. SI7†). To quantify aggregation propensity which is well known to significantly vary with concentration, we have also tested the capability of Ros87 to form amyloid fibers at higher concentrations. ThT assays carried out at a Ros87 concentration of 300 μM show a slightly faster fibril formation phenomenon, occurring after 26 hours, confirming the propensity of Ros87 to form amyloid fibrils under native conditions (Fig. SI8†).

We then evaluated the aggregation of Ml4_52–151_, a Ros87 iso-structural metal lacking homologue.^12^ DSC studies have shown that Ml4_52–151_ unfolds *via* a cooperative all-or-none transition starting at temperatures higher than 288 K and we therefore conducted aggregation studies at this temperature. We first followed the aggregation time courses of the Ml4_52–151_ protein by CD spectroscopy. The CD spectra (Fig. SI9† and 5b) acquired as a function of time show a small initial decrease of the secondary structure content after 60 hours. Accordingly, the size distribution measured *via* DLS shows the monomeric protein with a hydrodynamic radius of ∼2.3 nm and the appearance of larger aggregates of 291.4 ± 18 nm only after 168 hours. ThT analysis does not evidence any formation of amyloid aggregates within the first 168 hours (Fig. 5e). TEM confirmed these data, showing the absence of fibrils after 144 hours of incubation.

At the same temperature (288 K), when the protein concentration is 300 μM, the ThT assay shows the appearance of fibrils after 67 hours of incubation (data not shown). At 298 K, Ml4_52–151_ starts to form amyloid fibers after ∼50 hours (Fig. SI8†). This finding further supports the existence of a non-native precursor conformation for the amyloid formation accessed, when the unfolded state is significantly populated in solution, by thermal structural fluctuations.

We have also investigated the aggregation propensity of Ml1_53–149_. CD spectra of Ml1_53–149_ as a function of time, indicate that the protein slightly starts to lose the secondary structure content after about 50 hours (Fig. 5c and SI10E†). DLS analysis shows that protein aggregates start to form after 24 hours and ThT studies reveal only a small increase of fluorescence within the monitored 144 hours (Fig. 5c). TEM pictures illustrate the presence of amorphous aggregates after 25 hours, which remain structurally unperturbed for at least 144 hours, without conversion into amyloid fibrils (Fig. SI10A–D†). Accordingly, at 300 μM, Ml1_53–149_ starts to form amyloid aggregates after 18 hours of incubation (Fig. SI11†). Like Ros87, Ml1_53–149_ under acidic conditions is unfolded and shows, by means of DLS, a fast formation of oligomers (∼150 nm of diameter), which starts immediately without a well-defined lag phase.

## Discussion

Here, we present a study aiming to correlate the folding mechanism with amyloid formation propensity in highly homologous proteins. We therefore initially characterized the unfolding of Ros87, Ml1_53–149_ and Ml4_52–151_ by means of kinetics and equilibrium experiments. The results indicate that the three proteins significantly differ in terms of stability and (un)folding mechanisms. Particularly, Ros87 and Ml1_53–149_ appear much more stable to GdnHCl denaturation and are characterized by folding mechanisms including the presence of an intermediate. On the other hand, metal lacking Ml4_52–151_ folds according to a classic two-state model. This description is in agreement with a previous study^8^ based on equilibrium folding studies of Ros87 and Ml4_52–151_, which proposed that the metal coordination can intrinsically stabilize folding intermediates having a small energetic difference from the native state and therefore accessible by non-hierarchical multi-state transitions.

Successively, we have monitored the capability of Ros87 to form amyloid fibrils under native conditions and compared it to those of the two iso-structural proteins, Ml4_52–151_ and Ml1_53–149_. In particular, we show how, at a concentration of 100 μM, after 168 hours, amyloid formation of Ros87 has already started, while Ml1_53–149_ has formed only amorphous aggregates and Ml4_52–151_ is still monomeric in solution, though being thermally much less stable than Ros87 and Ml1_53–149_ (*T*_m_ = 306 K). Such amyloid fibril propensities have been confirmed by analogous studies performed at a protein concentration of 300 μM.

The central cores of the three globular structures (Fig. 1) have similar high tendencies to form aggregates (Fig. SI5†). This tendency is confirmed by the extremely fast aggregation behaviour of acidic Ros87 and Ml1_53–149_, *i.e.* in the absence of structural constraints that stabilize the native structure (Fig. SI7†) and by the behaviour of Ml4_52–151_ when incubated at 298 K (Fig. SI8†). Therefore, the three proteins share a very similar tertiary fold, which does not protect them from aggregation phenomena with similar efficacy, featuring significant differences in their aggregation propensities.

In fact, the presence of the metal ion and a Cys_2_His_2_ coordination sphere significantly affect the folding mechanism of Ros87 and, in turn, the conformational equilibria in which the native protein is involved. In particular, the existence of a folding intermediate, containing a β-hairpin nucleus stabilized by the metal binding, structurally and energetically close to the native state,^3^,^8^ could provide the precursor conformation prone to amyloid formation, as described in the “gain-of-interaction model” of the aggregation processes, a very restricted set of conformational changes that expose surfaces prone to polymerization otherwise inaccessible.^39^,^40^ Furthermore, the presence of a downhill folding mechanism, providing an ensemble of conformations that gain structures gradually till the native state is reached, further increases the number of protein conformational states prone to aggregation. This scenario, as shown by the aggregation behaviour of Ml1_53–149_, allows fast protein association, but does not seem to be *per se* sufficient to guarantee rapid amyloid fibril formation. On the other hand, the cooperative folding mechanism of Ml4_52–151_ prevents the protein to populate states susceptible to form intermolecular stable interactions that allow protein aggregation initiation. As a matter of fact, different from what was observed at 288 K, an efficient Ml4_52–151_ amyloid fibrillation is obtained at 298 K, when the globular protein is in equilibrium with a consistent fraction of the unfolded chain.^8^ These data further support the existence of a non-native precursor conformation for amyloid formation, which in the case of Ml4_52–151_, can be accessed by thermal structural fluctuations. Interestingly, the formation of amyloid fibrils by Ros87 is observed under experimental conditions comparable to those of *in vitro* model systems usually utilized to promote rapid amyloid formation.^41^

Amyloid fibril formation by globular proteins under native conditions is a crucial phenomenon at the basis of the pathogenesis of relevant protein deposition diseases. Since every protein sequence is, in principle, prone to form stable intermolecular aggregates,^1^ protein evolution has preferred sequences able to fold cooperatively so as to prevent the formation of “prone to aggregation” conformational states. Nonetheless, precursor states promoting aggregation may be accessible when native-like conformations are reached through thermal structural fluctuations. In this study, we show that folding mechanisms when influenced by metal recruitment may induce the stabilization of native-like metal binding conformational states that sensibly increase protein fibrillation phenomena. These findings may also have interesting implications in evolutionary terms: the presence of domains that may provide flexibility at the expense of cooperativity in folding pathways is an essential prerequisite for downhill folders.^42^

Therefore, although characterized by an increased tendency to aggregate, downhill folders could be ancestral starting points by which evolution could select new protein functions while exploring large conformational ensembles. This role is possibly even more relevant in metal binding proteins in which the metal recruitment may stabilize an intermediate from which a variety of final conformations can be reached *via* a downhill pathway. As a matter of fact, Ros87 metal sites have been proposed to be the ancestral domain from which the prokaryotic zinc finger family has evolved^43^ which has been possibly exploited by means of such a kind of evolutionary conformation selection. Overall, this study, underlining the relevance of non-native partially unfolded states in the aggregation process, shows how metal binding can influence the folding pathway of relatively small domains and thereby control conformational accessibility to aggregation-prone states, which in turn changes aggregation kinetics. While the reported model domains have little direct disease-relevance, our study shows how a deeper knowledge of metal recruitment in metal binding proteins also related to their aggregation behaviour will help to shed light on the role of metal ions in the development of protein deposition diseases.

## Statement of contribution

Gianluca D'Abrosca, Maddalena Palmieri, Luigi Russo, Valeria Sivo, Ilaria Baglivo, Michele F. M. Sciacca Angelo Toto and Luciano Pirone produced and purified samples and performed all the spectroscopic and kinetic experiments and analysed the data.

Rosarita Tatè and Roksana Majewska performed and analysed TEM experiments.

Paolo V. Pedone, Carla Isernia, Mario De Stefano, Emilia M. Pedone, Massimo Coletta and Stefano Gianni designed the experiments and supervised the study.

Gaetano Malgieri, Danilo Milardi and Roberto Fattorusso conceived and designed the study and wrote the manuscript.

## Conflicts of interest

There are no conflicts to declare.

## Supplementary Material

Supplementary information
