# Supplementary material for: Folding mechanisms steer the amyloid fibril formation propensity of highly homologous proteins
Source: Chem Sci. 2018 Mar 1;9(13):3290–8. doi: 10.1039/c8sc00166a (PMC5933289; doi:10.1039/c8sc00166a)
Supplement: Supplementary file 1 [file SC-009-C8SC00166A-s001.pdf]

Electronic Supplementary Material (ESI) for Chemical Science.
This journal is © The Royal Society of Chemistry 2018

Folding mechanisms steer amyloid fibrils formation propensity of highly homologous proteins

Gaetano Malgieri[1], Gianluca D'Abrosca[1], Luciano Pirone[2], Angelo Toto[3], Maddalena Palmieri[1#], Luigi Russo[1], Michele Francesco Maria Sciacca[4], Rosarita Tatè[5], Valeria Sivo[1], Ilaria Baglivo[1], Roksana Majewska[1§], Massimo Coletta[6], Paolo Vincenzo Pedone[1], Carla Isernia[1], Mario De Stefano[1], Stefano Gianni[3], Emilia Maria Pedone[2], Danilo Milardi[4*] and Roberto Fattorusso[1*]

1: Department of Environmental, Biological and Pharmaceutical Sciences and Technologies, University of Campania "Luigi Vanvitelli", Via Vivaldi 43, 81100 Caserta (Italy).

2: Institute of Biostructures and Bioimaging - CNR, Via Mezzocannone 16, 80134 Naples (Italy).

3: Department of Biochemical Sciences "Alessandro Rossi Fanelli" - University of Rome "La Sapienza", Piazzale Aldo Moro 5, 00185, Roma (Italy).

4: Institute of Biostructures and Bioimaging - CNR, Viale A. Doria 6, 95125 Catania (Italy).

5: Institute of Genetics and Biophysics "Adriano Buzzati-Traverso" - CNR, Via P. Castellino 111, 80131 Napoli (Italy).

6: Department of Clinical Sciences and Translational Medicine, University of Rome "Tor Vergata", Via Montpellier 1, 00133, Roma (Italy).

*Correspondence: roberto.fattorusso@unicampania.it, dmilardi@unict.it.

Present Addresses:

[#] Patheon via Morolense 5, 03013 Ferentino (Fr) - Italy.

[§] Unit for Environmental Sciences and Management- School of Biological Sciences- North-West University - Private Bag X6001- Potchefstroom- South Africa AND South African Institute for Aquatic Biodiversity (SAIAB) - Private Bag 1015 - Grahamstown 6140- South Africa.

Supplementary Information

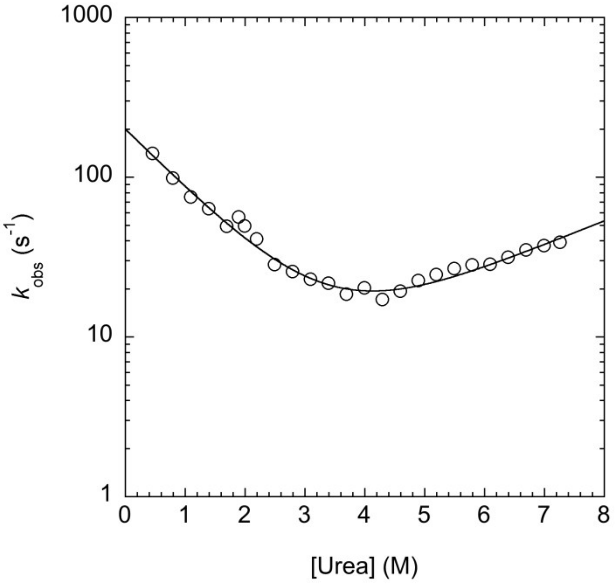

FIGURE SI1 – Chevron plot of $Ml4_{52-151}$ in Urea

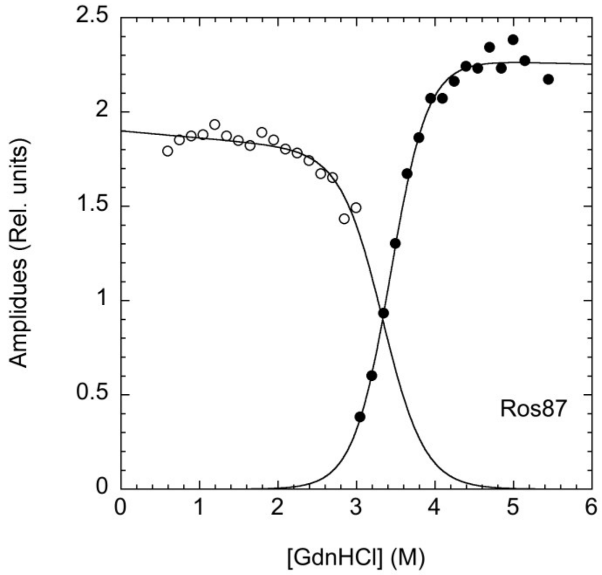

FIGURE SI2 – Folding and unfolding kinetic transitions of Ros87. Open and filled circles refer to the amplitudes of the kinetic traces observed in the kinetic refolding and unfolding experiments respectively.

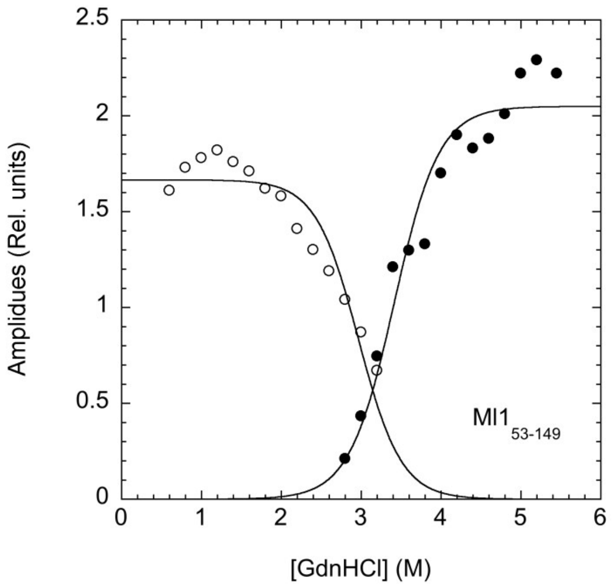

FIGURE SI3 – Folding and unfolding kinetic transitions of Ml1$_{53\text{-}149}$

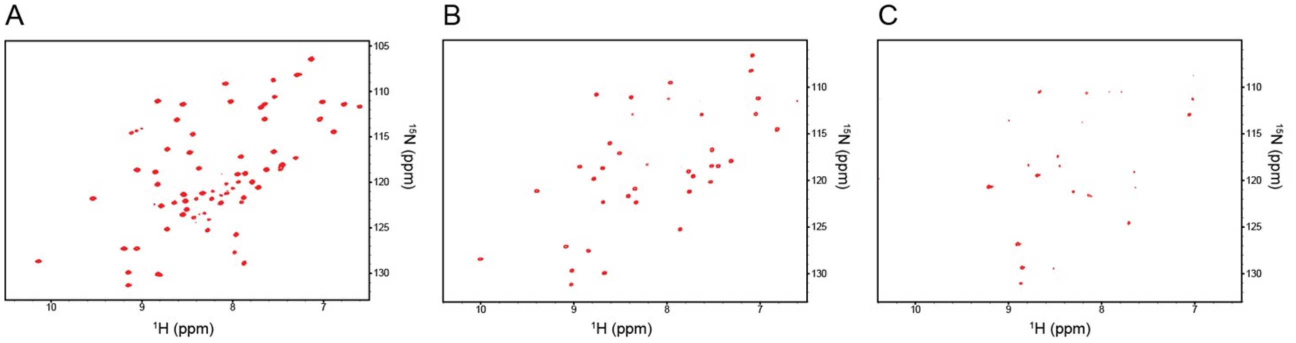

FIGURE SI4 – Unfolding of Ml1$_{53\text{-}149}$ by NMR: ^{1}H-^{15}N-HSQCs of the protein at 298K (A), 323K (B) and at 353K (C). NMR samples contained 0.8 mM ^{15}N- Ml1$_{53\text{-}149}$, 20 mM phosphate buffer, 0.2 M NaCl, 4 mM TCEP, pH 6.8, and 90% H_2O/10% $^{2}H_2O$. The experiments were acquired on a Varian Unity INOVA 500 MHz spectrometer. Temperature-induced chemical shift perturbations have been monitored in a series of ^{1}H−^{15}N HSQC spectra acquired in a range from 278 to 353 K at regular intervals of temperature. The resonances exhibit a continuous chemical shift variation indicating a fast protein folding process and some of them still preserved a good spectral dispersion at 353 K. The presence of visible cross-peaks up to 353 K is in agreement with the mechanism of folding described by the other techniques.

```
Ml1 53-149    1~~SAAKPE~ALEPA VPIRKSVTPD YIICLDDGKK FKSLKRHLST AKWHLPADYP MVAPNYAAAR SALAKTMGLG RKPKEPEART RKKAAA~97
Ml4 52-151    1GRPAENP~~VLTPA VNPKKSVFPD YIVSLEDGRK FKSMKRHLGL TKWDLPRDYP MVAPNYAATR SALAKASGLG RKAAPVKKAP AKRKAKA100
Ros87         1~~~~AVNVEKQKPA VSVRKSVQDD HIVCLECGGS FKSLKRHLTT EKWDLPVDYP MVAPAYAEAR SRLAKEMGLG QRRKANR~~~ ~~~~~~~87
```

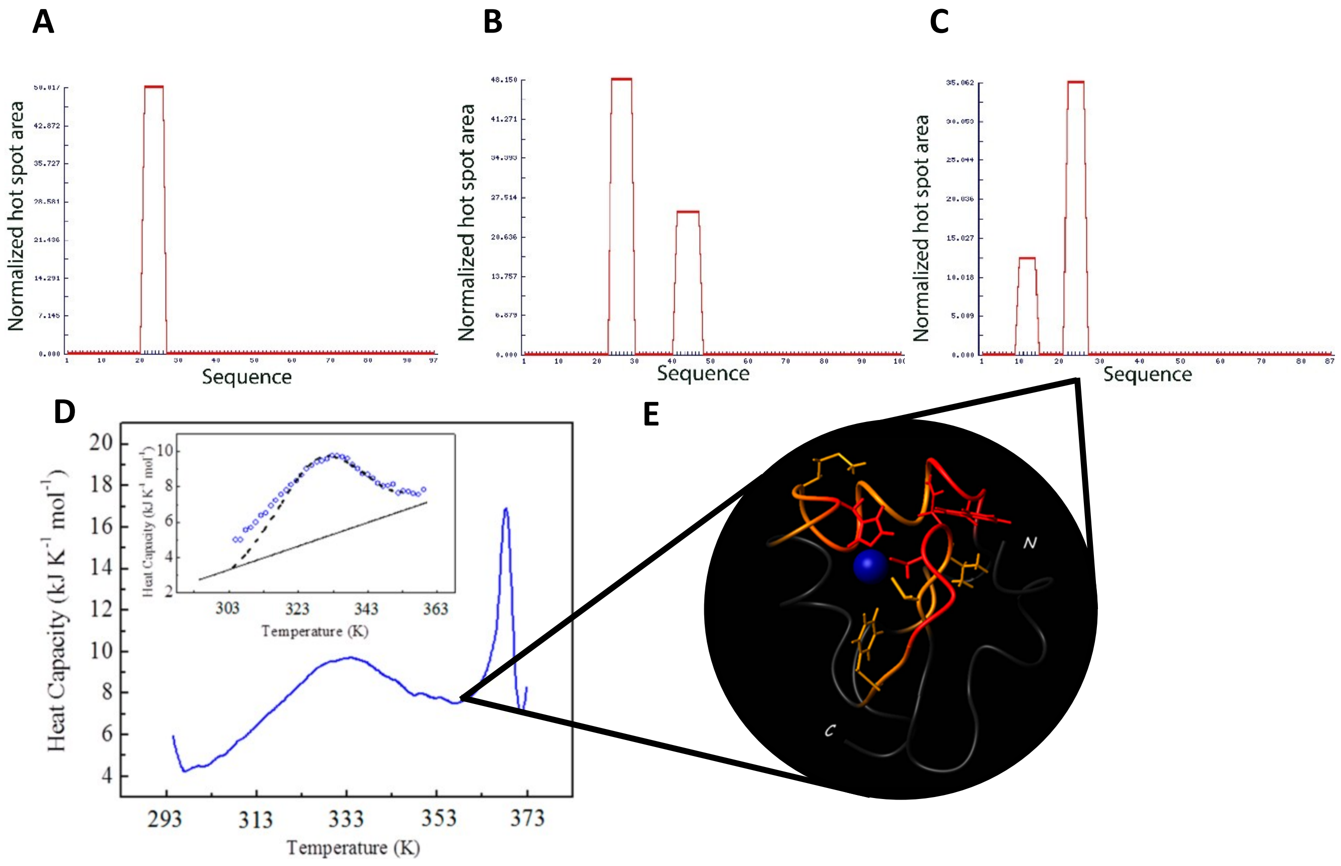

FIGURE SI5 - Alignment of the three proteins: the amino acid stretches with high propensity to aggregate are in red; AGGRESCAN results for Ros87 (C), $Ml4_{52–151}$ (B) and $Ml1_{53-149}$ (A); thermal unfolding of Ros87 followed by DSC (D); Ros87 zinc containing intermediate present at 353K (Palmieri *et al.*, JACS 2013).

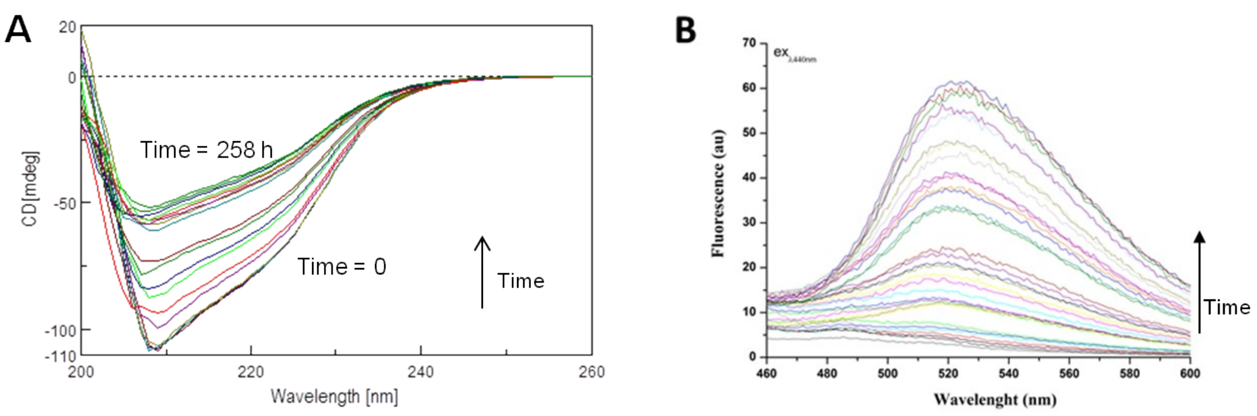

Figure SI6 - (**A**) CD spectra of Ros87 incubated at 298K recorded every 12 hours for 10 days. (**B**) ThT florescence assay of Ros87 incubated at 298K: spectra were recorded every 12 hours.

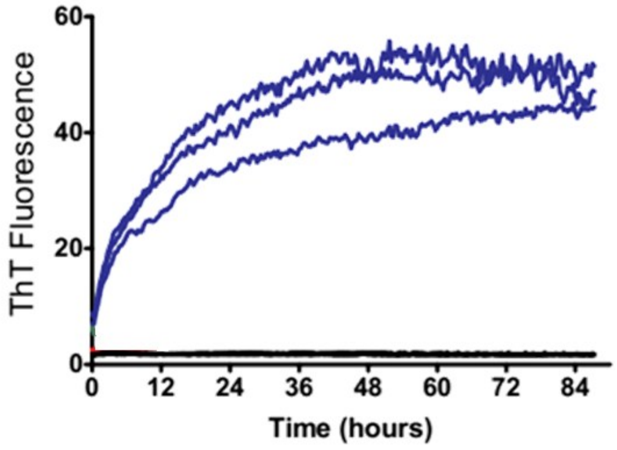

FIGURE SI7 – Aggregation behavior of unfolded Ros87 in acidic conditions (pH=3) at 298K followed by Thioflavin T fluorescence, recorded as a triplicate.

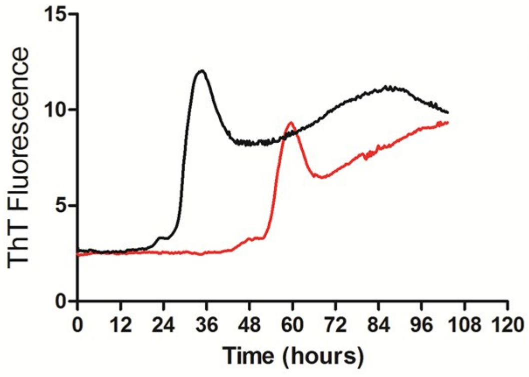

FIGURE SI8 - Aggregation behavior of Ros87 (black) and Ml4$_{52-151}$ (red) at 300 μM and 298K followed by Thioflavin T fluorescence.

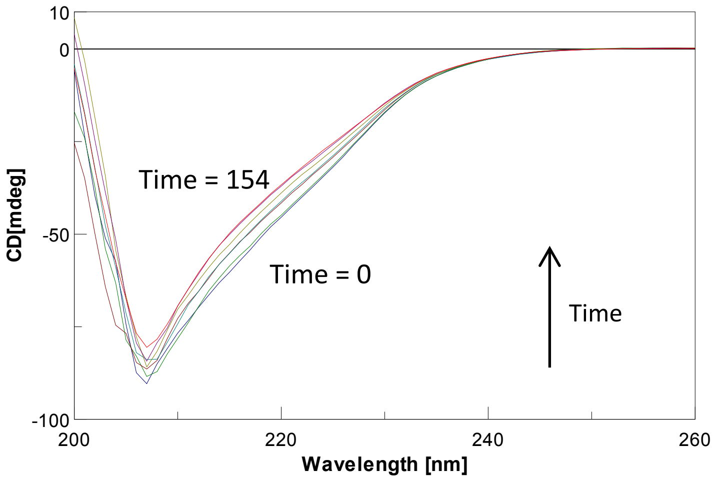

FIGURE SI9 - CD spectra of Ml4$_{52-151}$ incubated at 288K recorded every 24 hours for 154 hours.

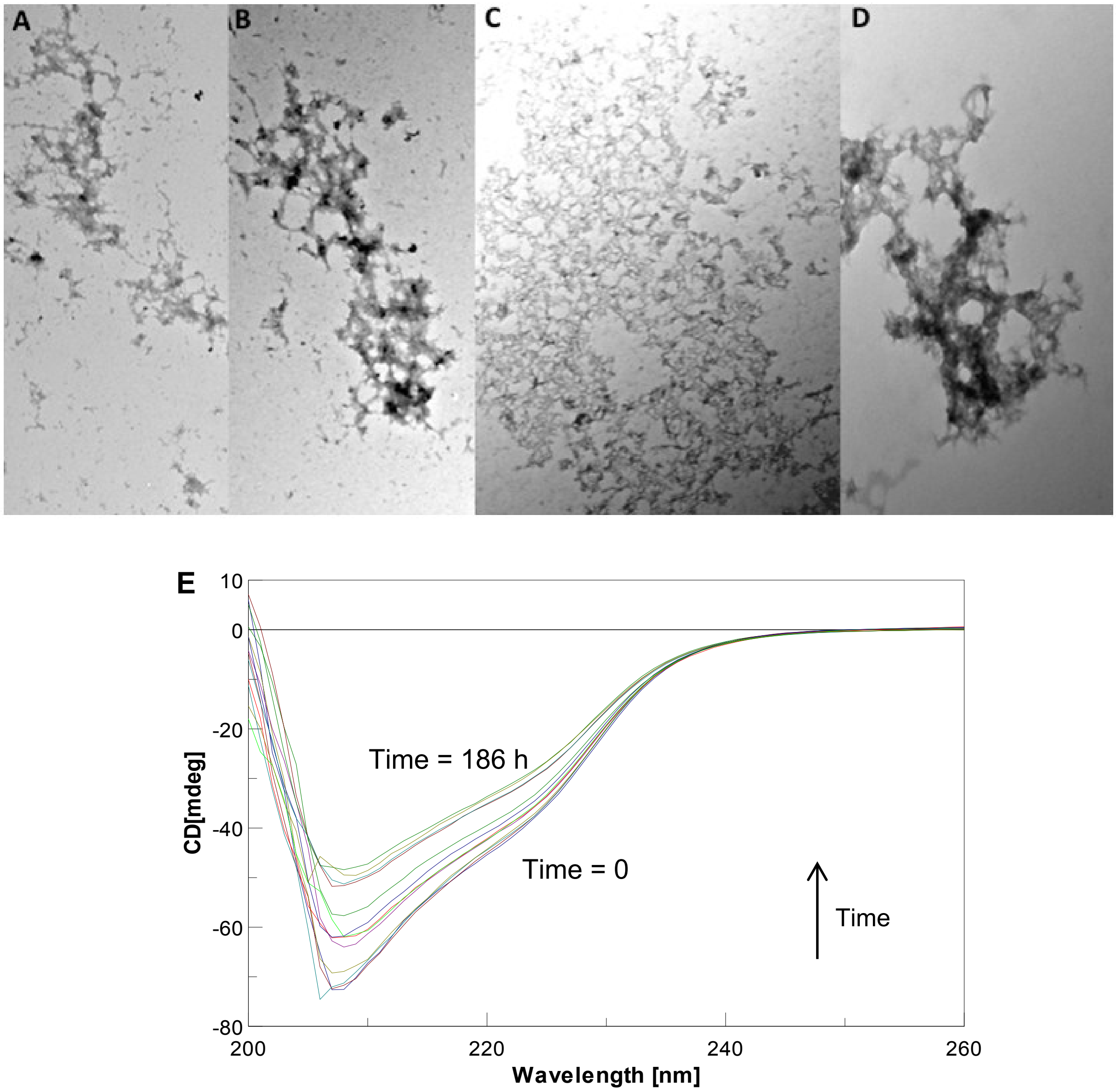

Figure SI10 - Aged Ml1$_{53-149}$: amorphous aggregates (panels A-D) - CD spectra of Ml1$_{53-149}$ incubated at 298K recorded every 12 hours for 186 days (panel E).

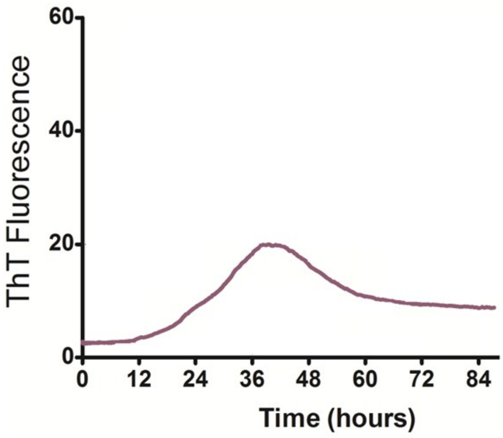

FIGURE SI11 - Aggregation behavior of Ml1$_{53-149}$ at 300 μM and 298K followed by Thioflavin T fluorescence.
